# Impact of Emergent Cervical Carotid Stenting in Tandem Occlusion Strokes Treated by Thrombectomy: A Review of the TITAN Collaboration

**DOI:** 10.3389/fneur.2019.00206

**Published:** 2019-03-11

**Authors:** François Zhu, Serge Bracard, René Anxionnat, Anne-Laure Derelle, Romain Tonnelet, Liang Liao, Gioia Mione, Lisa Humbertjean, Jean-Christophe Lacour, Gabriela Hossu, Mohammad Anadani, Sébastien Richard, Benjamin Gory

**Affiliations:** ^1^Department of Diagnostic and Therapeutic Neuroradiology, University Hospital of Nancy, Nancy, France; ^2^INSERM U1254, IADI, University of Lorraine, Nancy, France; ^3^Department of Neurology, Stroke Unit, University Hospital of Nancy, Nancy, France; ^4^Department of Neurosurgery, Medical University of South Carolina, Charleston, SC, United States; ^5^Centre d'Investigation Clinique Plurithématique, INSERM U1116, University Hospital of Nancy, Vandoeuvre-lès-Nancy, France

**Keywords:** tandem occlusion, stroke, carotid stenting, endovascular treatment, thrombectomy, emergent stenting in tandem occlusion

## Abstract

**Introduction:** Endovascular therapy has been shown to be an effective and safe treatment for tandem occlusion. The endovascular therapeutic strategies for tandem occlusions strokes have not been adequately evaluated and the best approach is still controversial. The TITAN (Thrombectomy in TANdem occlusions) registry was a result of a collaborative effort to identify the best therapeutic approach for acute ischemic stroke due to tandem lesion. In this review, we aim to summarize the main findings of the TITAN study and discuss the challenges of treatment for tandem occlusion in the era of endovascular thrombectomy.

**Methods:** A review of the data from the multicenter international observational and non-randomized TITAN registry was performed. The TITAN registry included acute ischemic stroke patients with tandem lesions (proximal intracranial occlusion and cervical carotid artery occlusion or stenosis>90%) who were treated with thrombectomy with or without carotid artery stenting.

**Results:** Prior intravenous thrombolysis and emergent cervical carotid stenting were associated with higher reperfusion (mTICI 2b-3 and mTICI 3) rates at the end of the intervention. Poor outcome did not occur more frequently after stenting than after conservative treatment of the cervical carotid lesion. Emergent carotid stenting with antithrombotic agents and intracranial thrombectomy yielded higher reperfusion rate and good outcome (90 day mRS 0–2) compared to other strategies (carotid artery stenting and thrombectomy without antithrombotic, angioplasty and thrombectomy, or thrombectomy alone). Pretreatment intravenous thrombolysis was not associated with increased risk of hemorrhagic complications. Likewise, periprocedural unfractionated heparin did not modify the efficacy and safety results. Etiology of carotid artery lesion (atherosclerosis vs. dissection) did not emerge as predictor of outcome or recanalization.

**Conclusion:** Emergent stenting of the cervical carotid lesion with antithrombotic agents in conjunction to thrombectomy appears to be the best treatment strategy for acute ischemic strokes with tandem lesions. These findings will be further investigated in the ongoing randomized controlled TITAN trial.

## Introduction

Tandem occlusions, defined as proximal anterior circulation intracranial occlusion and an ipsilateral cervical internal carotid artery (ICA) high-grade stenosis or occlusion, account for about 10–15% of acute ischemic strokes due to large-vessel occlusions (LVO). Data on the outcome of endovascular treatment of tandem occlusion is scarce (1), and tandem occlusion was among the exclusion criteria of most of the endovascular randomized trials (2, 3). Treatment effect of mechanical thrombectomy (MT) in patients with tandem occlusions was comparable to isolated intracranial occlusion in the HERMES meta-analysis (3); however, the optimal revascularization strategy of the extracranial lesion was not reported and many patients with non-severe stenosis (<70–80%) were included, especially in the MR CLEAN trial (4). Emergent carotid artery stenting in conjunction with intracranial MT is one of the therapeutic approaches for tandem occlusions. However, the safety of this approach is uncertain mainly due to the need for peri-procedural antithrombotic agents, which could increase the risk of hemorrhagic complications. In this review, we aimed to summarize the results of the TITAN study, focusing on the efficacy and safety of emergent carotid artery stenting in conjunction with mechanical thrombectomy approach, and to compare our results to the recent literature.

## Methods

### The TITAN Collaboration

The TITAN (Thrombectomy In TANdem lesions) international collaboration pooled individual data of non-randomized thrombectomy databases from 18 comprehensive stroke centers for all consecutive anterior circulation tandem patients who underwent emergent thrombectomy between January 2012 and September 2016. Patients were included if they presented with acute ischemic stroke due to anterior circulation tandem occlusion and were treated with modern endovascular devices such as second-generation stent retriever and/or contact aspiration. Tandem lesion was defined as a proximal intracranial occlusion (distal ICA, and/or M1 and/or M2 segment of the middle cerebral artery) and an extracranial ICA lesion (complete occlusion or stenosis ≥90% North American Symptomatic Carotid Endarterectomy Trial).

Treatment of the extracranial ICA lesion was left at the discretion of the operators. Four therapeutic strategies for the cervical steno-occlusive lesion were possible: (1) acute stenting with antithrombotic agents, (2) acute stenting without antithrombotic agents, (3) acute balloon angioplasty without stenting, and (4) no treatment. Intravenous thrombolysis was administered for eligible patients according to the international guidelines. Intravenous sedation and general anesthesia were permitted.

All patients included had a computed tomography or magnetic resonance imaging at 24 h after treatment onset to assess for hemorrhagic complications. The local ethics committees approved the use of patient data for the retrospective analysis and waived the need for patient consent. All data were retrospectively collected and scored by one neuro-interventionist in each center.

### Outcomes

All studies primary outcome was the good outcome defined as a 90 day modified Rankin Scale (mRS) 0–2. Secondary outcomes included successful reperfusion defined as modified Thrombolysis In Cerebral Infarction (mTICI) score 2b-3 (5), complete reperfusion (mTICI 3), all-cause mortality at 90 day, any procedural related complications, any intracranial hemorrhage (ICH), and symptomatic ICH. Cerebral hemorrhages were classified as hemorrhagic infarction (HI) or parenchymal hematoma (PH) according to the European Cooperative Acute Stroke Study II criteria (6). Symptomatic ICH (sICH) was defined as any parenchymal hematoma, subarachnoid hemorrhage, or intraventricular hemorrhage associated with worsening of the National Institutes of Health Stroke Scale (NIHSS) score by 4 points or more.

## Results

### Cervical ICA Lesion Etiology

Among the 295 included patients, the etiology of the cervical ICA lesion was atherosclerosis in 230 (78%) patients and dissection in 65 (22%) patients (7). Interestingly, the etiology of the cervical ICA lesion (atherosclerosis vs. dissection) did not impact the final reperfusion rates or clinical outcomes (7). This finding suggests that the etiology of the extracranial disease should not influence the treatment strategy.

### Treatment of the Cervical ICA Lesion Efficacy

Papanagiotou et al. compared four therapeutic strategies of the cervical steno-occlusive lesion (8). Treatment of the cervical ICA lesion in conjunction with intracranial mechanical thrombectomy led to a higher rate of successful reperfusion compared to thrombectomy without ICA treatment (79.4 vs. 60.2%; OR = 2.04; 95% CI = 1.18–3.51; *P* = 0.011), with or without prior thrombolysis. Although was not statistically significant, the rate of good outcome (90 day mRS 0-2) was higher in patients who received cervical ICA treatment with intracranial mechanical thrombectomy compared to those who received mechanical thrombectomy alone without ICA lesion treatment (53.4 vs. 41.0%; OR = 1.42; 95%CI = 0.83–2.41; *P* = 0.20) (8). In addition, there was no significant difference in the rate of 90 day mortality (11.4 vs. 17.1%; OR = 0.56; 95%CI = 0.29–1.09; *P* = 0.086), or sICH (4.6 vs. 4.6%; OR = 1.23; 95%CI = 0.40–3.72; *P* = 0.72) between the two groups after pre-specified adjustments (8).

#### Reperfusion Outcomes After Emergent Stenting

A significantly higher rate of successful reperfusion in the carotid stenting and antiplatelet group compared to the thrombectomy alone group was observed (83.1 vs. 60.2%; OR = 2.66; 95% CI = 1.38–5.10; *P* = 0.003) (Table 1) (8). Likewise, complete reperfusion rate was higher in the former group (41.2 vs. 23.2%; OR = 1.91; 95% CI = 1.08–3.40; *P* = 0.026). In addition, carotid stenting with antithrombotic appeared superior to stenting without antithrombotic and balloon angioplasty groups, although these differences were not statistically significant (8).

**Table 1:** Efficacy and safety outcomes between patients treated by intracranial thrombectomy alone vs. those treated by intracranial thrombectomy and cervical carotid artery stenting with antiplatelets [from Papanagiotou et al. (8)].

**Outcomes**	**Intracranial thrombectomy and cervical ICA stenting with antiplatelets (*n* = 256)**	**Intracranial thrombectomy alone (*n* = 108)**	**Unadjusted analysis**		**Adjusted analysis**^**^a^**^	
			**OR (95%CI)**	***P***	**OR (95%CI)**	***P***
mTICI 2b-3	212/255 (83.1)	65/108 (60.2)	3.26 (1.96–5.41)	<0.001	2.66 (1.38–5.10)	0.003
mTICI 3	105/255 (41.2)	25/108 (23.2)	2.32 (1.39–3.88)	0.001	1.91 (1.08–3.40)	0.026
90-day mRS 0-2	147/254 (57.9)	43/105 (41.0)	1.98 (1.24–3.15)	0.004	1.44 (0.77–2.67)	0.25
90-day mortality	24/254 (9.5)	18/105 (17.1)	0.50 (0.26–0.98)	0.042	0.44 (0.21–0.94)	0.033
Symptomatic intracerebral hemorrhage	13/255 (5.1)	5/108 (4.6)	1.11 (0.38–3.18)	0.85	1.24 (0.36–4.23)	0.73

*Values are expressed as no/total no. (%) unless otherwise indicated. ORs were calculated using patients treated by intracranial thrombectomy alone as reference group*.

a *Adjusted for prespecified confounders (age, baseline NIHSS score, and prior IV thrombolysis) and center (including as random effect)*.

*Symptomatic intracerebral hemorrhage was defined as any parenchymal hematoma, subarachnoid hemorrhage, or intraventricular hemorrhage associated with worsening of the NIHSS score of 4 points or more according to the ECASS-2 criteria*.

*NIHSS, National Institutes of Health Stroke Scale; IV, intravenous; mTICI, modified Thrombolysis In Cerebral Infarction; mRS, modified rankin score*.

#### Clinical Outcomes After Emergent Stenting

Similar results were found for clinical outcomes at 90 days with a higher rate of good outcome in the setting of ICA stenting with antithrombotic agents compared to thrombectomy alone (57.9 vs. 41.0%; unadjusted OR = 1.98; 95% CI = 1.24–3.15; *P* = 0.004) (8). However, this difference became non-significant after adjusting for pre-specified confounders (OR = 1.44; 95% CI = 0.77–2.67; *P* = 0.25) (Table 1). In addition, good outcome was achieved more in the stenting with antithrombotic group than the stenting without antithrombotic, and balloon angioplasty groups; however, the difference did not reach statistical significance which likely related to a small sample size in the latter groups (8).

### Safety of Emergent Carotid Stenting

#### Mortality

In case of acute stenting, mortality within 90 days was significantly lower (9.5 vs. 17.1%; OR = 0.50; 95% CI = 0.26–0.98; *P* = 0.033) compared to intracranial thrombectomy alone (8). Predictors of 90 day mortality in the TITAN cohort were age (OR = 1.05; 95% CI = 1.02–1.08; *P* = 0.001), current smoker (OR = 1.05; 95% CI = 1.02–1.08; *P* = 0.001), NIHSS scores (OR = 1.14; 95%CI = 1.07–1.22; *P* < 0.001), ASPECTS scores (OR = 0.75; 95% CI = 0.62–0.89; *P* = 0.001), and prior thrombolysis (OR = 0.48; 95% CI = 0.25–0.90; *P* = 0.022) (8).

#### Intracranial Hemorrhagic Transformation

**Figure 1:**
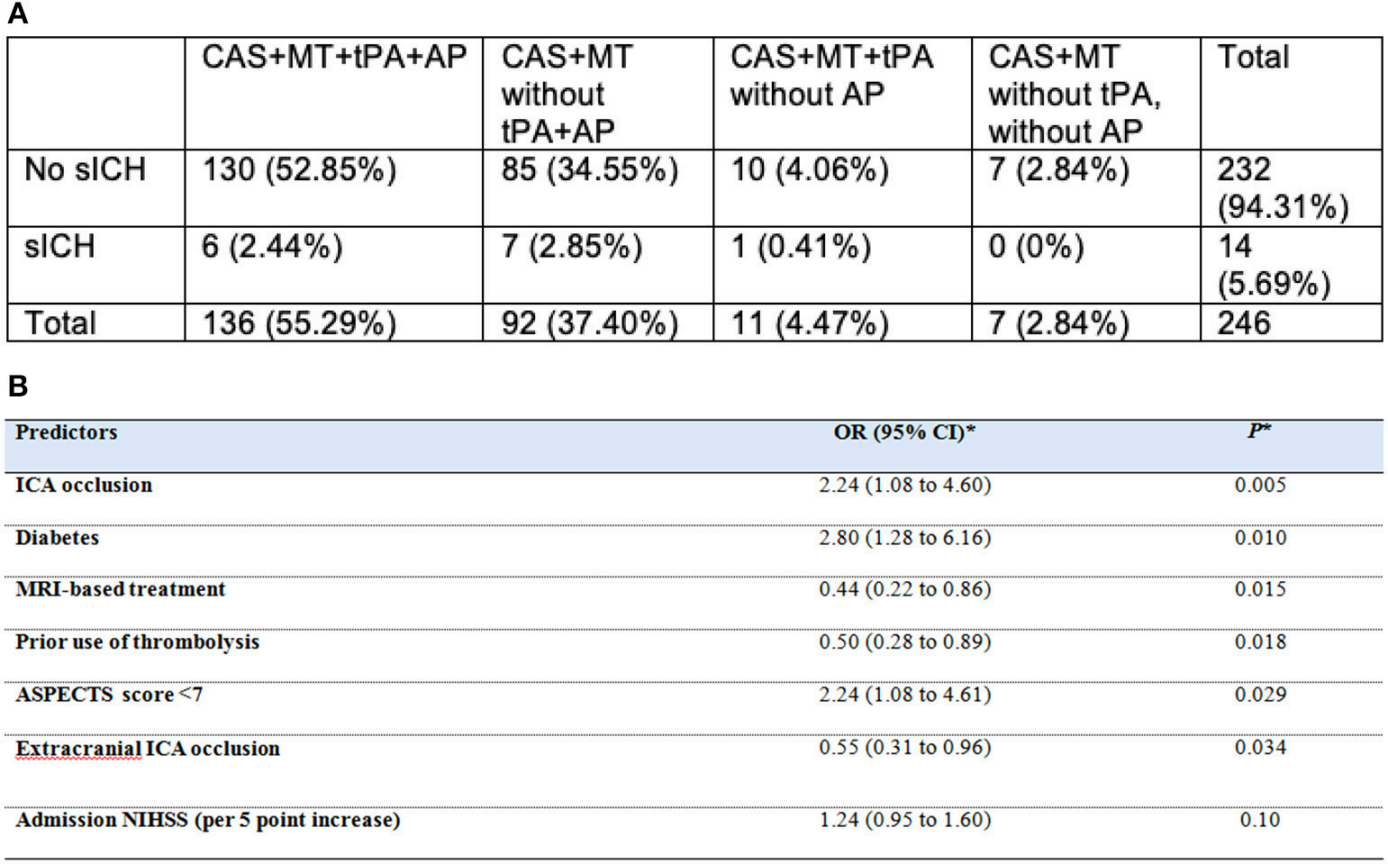
**(A)** sICH prevalence according to the endovascular and pharmacological strategy. CAS, Carotid Acute Stenting; MT, Mechanical Thrombectomy; tPA, IV thrombolysis; AP, Periprocedural use of antiplatelet. **(B)** Multivariable regression analysis of predictors of any hemorrhagic transformation (from Zhu et al. (9)). ^*^Calculated after handling missing data by multiple imputations using a backward-stepwise logistic model including all univariate predictors at *P* < 0.20. ASPECTS, Alberta Stroke Program Early CT Score; CI, confidence interval; ICA, internal carotid artery; IV, intravenous; MRI, magnetic resonance imaging; NIHSS, National Institutes of Health Stroke Scale; OR, odds ratio.

Of the 267 patients with available data on hemorrhagic transformation, there were 66% (24.7%) of HI (31 HI1 and 35 HI2) and 38 (14.2%) of PH (24 PH1 and 14 PH2) (9). Intracranial and extracranial ICA occlusions, diabetes mellitus, no prior use of IVT were predictors of hemorrhagic transformation (9). Interestingly, no impact of thrombolysis on different outcomes was observed in our study. Administering one or even two antiplatelet drugs was associated with a lower risk of sICH (Figure 1A) (9).

### Pharmacological Adjuvant Therapy Strategy

In TITAN, periprocedural heparin use during endovascular therapy of patients with tandem occlusion was not associated with a better clinical outcome or a higher risk of intracerebral hemorrhagic complications (Zhu et al., submitted). Acute ICA stenting and antiplatelet administration were not predictors of HI and PH within 24 h in TITAN registry (Figure 1B) (9).

### Impact of Prior Intravenous Thrombolysis

We found an association between intravenous thrombolysis (IVT) and successful reperfusion when we included all patients in the TITIAN registry regardless of the ICA treatment strategy (OR, 1.47; 95% CI, 1.01–2.12) (10). Interestingly, when only patients treated with carotid artery stenting included, we did not find association between IVT and functional outcome or more importantly safety outcomes (11).

### Which Occlusion to Treat in First?

Among the patients from the TITAN cohort, order of treatment of intracranial occlusion or cervical occlusion led to a similar rate of successful reperfusion (12). As expected, treating the intracranial occlusion first was associated with faster time from puncture to reperfusion; however, the order of treatment was not associated with clinical outcomes (12).

## Discussion

The main result of the TITAN collaboration is that mechanical thrombectomy and emergent carotid stenting with antiplatelet therapy seems to be the most effective therapeutic approach for tandem lesions in acute ischemic stroke, even in patients who received prior intravenous thrombolysis. A similar association between acute stenting and successful reperfusion in stroke patients with tandem lesion treated with thrombectomy was reported by Sadeh-Gonik et al. in a meta-analysis including a total of 590 patients pooled from 13 case series (13).

The rate of hemorrhagic complications after tandem occlusion endovascular treatment seems similar to the rate of hemorrhagic complications after isolated intracranial MT. In the aforementioned meta-analysis, the reported rates of all hemorrhage and sICH were 24 and 8%, respectively (13), whereas the rate of HI was 23% and the rate of PH was 13% in THRACE (THRombectomie des Artères CErebrales) trial (2). Similar safety outcomes for acute ICA stenting were found by Behme et al. in a German multicenter study (14). Our results parallel those from STRATIS (Systematic Evaluation of Patients Treated With Neurothrombectomy Devices for Acute Ischemic Stroke) registry, which included 147 patients with tandem occlusions, treated with Solitaire device with or without carotid artery stenting (15).

Despite the encouraging results of the abovementioned studies demonstrating high successful reperfusion rate and good outcome with carotid artery stenting, the need of antithrombotic agents in the acute phase makes it a less appealing approach prompting some experts to recommend alternative approaches such as MT alone or angioplasty without acute stenting (16). Two retrospective studies demonstrated a high successful reperfusion rate and good outcome with angioplasty or MT alone (17–19); however, these studies suffered a small sample size. With angioplasty or MT alone approach, antithrombotic agents are often not required; therefore, the risk of hemorrhagic transformation is of lesser concern. As a result of inconsistent literature and lack of consensus, the best strategy remains unknown highlighting the need for randomized controlled trial. These precautionary approaches obviate the theoretical hemorrhagic risks due to the antiplatelets administration. Otherwise, these analyses did not take into account cervical occlusion as treatment of extracranial disease, which appears to be effective to prevent early embolic recurrence (20) and better reperfusion rates. Then, should we consider acute carotid stenting as the best strategy? And if yes, is “the best is the enemy of the good”? Actually no deleterious effect of carotid stenting has been already proved (19). To date, as Blassiau et al. said, choice of strategy “may vary between institution and physicians and adapts as a result of personal experience” (17), though the absence of consensus. This debate highlights the need of a randomized controlled trial for tandem lesions population.

Bridging with intravenous thrombolysis prior to thrombectomy remains the standard of care in eligible patients (21). Bridging approach was repeatedly shown to be superior to MT alone (22, 23). TITAN results are in line with previous studies and showed that intravenous thrombolysis prior to endovascular therapy is safe and may be helpful in patients with tandem occlusions.

We learnt from the cardiology literature that antiplatelet therapy is essential in case of stenting to prevent in-stent thrombosis. In case of acute stroke, the decision on best antithrombotic treatment is more complex, mainly due to the risk of hemorrhagic complications. Despite the extensive literature addressing the best long-term antiplatelet therapy (24), the best antiplatelet therapy in the acute phase following endovascular therapy is unknown; especially in case of carotid artery stenting. A recent systematic review found possible increased risk of sICH with periprocedural use of antiplatelets with neutral effect on the functional outcome (25).

In the TITAN study, we did not find an increased risk of hemorrhage with periprocedural antithrombotic use; on the contrary, patients who were treated with carotid stenting and antithrombotic agents performed better than those who did not receive periprocedural antithrombotic agents.

Heparin is commonly used in endovascular therapy and was found to be associated with good outcome in a *post-hoc* analysis of TREVO-2 trial (26). We did not find a significant association between periprocedural heparin use and clinical or safety outcomes. Heparin use theoretically reduces the risk of re-occlusion and distal emboli during endovascular therapy (27); unfortunately, the data regarding re-occlusion or distal emboli was not available in the TITAN registry, which may explain the lack of benefit of heparin in our registry. The ongoing randomized controlled trial MR CLEAN MED (Multicenter Randomized Clinical trial of Endovascular treatment for Acute ischemic stroke in the Netherlands investigating the effect of periprocedural MEDication) (28) will further evaluate the risks and benefit of periprocedural antithrombotic with aim to identify the best periprocedural medications.

Concerning the order to treat, treating the cervical lesion first “neck-first” provides accessibility to the distal occlusion, whereas treating the intracranial occlusion first “head-first” has advantage of faster reperfusion, but theoretically increase the risk of distal emboli. Studies comparing the two approaches provided conflicting results. Meta-analysis found no difference between the two approaches (19); on the contrary, a retrospective analysis of 171 tandem stroke patients found that patients who were treated with “head-first” approach performed better than those treated with “neck-first” approach (29).

Despite the large sample size and multi-center design, the TITIAN registry suffered from multiple limitations. First, the observational and non-randomized design has known methodological shortcomings. Second, there was no core-lab assessment of brain imagings, which may have introduced bias to our results. Third, despite handling the missing data with multiple imputations, the risk of bias is still a concern. Finally, treatment strategy was left to the interventionist discretion, and drug dosages and administration times were unknown, which likely subject to selection bias.

## Conclusion

Emergent stenting of the cervical carotid artery steno-occlusion lesion with periprocedural antithrombotic agents and intracranial mechanical thrombectomy was associated with favorable clinical and radiological outcomes. No safety concerns were reported in the TITAN collaboration, especially concerning hemorrhagic transformation risks. However, due to several discordances in the literature, further randomized controlled trials are warranted, as the on-going French multicenter prospective randomized TITAN trial.

## Author Contributions

FZ and BG substantial contribution to the conception and design of the work, drafting the work, final approval of the version to be published and agreement to be accountable for all aspects of the work in ensuring that questions related to the accuracy or integrity of any part of the work are appropriately investigated and resolved. SB, RA, A-LD, RT, LL, GM, LH, J-CL, GH, MA, and SR substantial contributions to the interpretation of data for the work, revising the work critically for important intellectual content, final approval of the version to be published and agreement to be accountable for all aspects of the work in ensuring that questions related to the accuracy or integrity of any part of the work are appropriately investigated and resolved.

### Conflict of Interest Statement

The authors declare that the research was conducted in the absence of any commercial or financial relationships that could be construed as a potential conflict of interest.
